# Standardization of medical service indicators: A useful technique for hospital administration

**DOI:** 10.1371/journal.pone.0207214

**Published:** 2018-11-28

**Authors:** Li Wu, Conghua Ji, Hanti Lu, Xuewen Hong, Shan Liu, Ying Zhang, Qiushuang Li, Sijia Huang, Penglei Zhou, Jiong Yao, Yuxiu Hu

**Affiliations:** 1 Center of Clinical Evaluation, Zhejiang Provincial Hospital of Traditional Chinese Medicine, Hangzhou, Zhejiang, China; 2 Department of Information, Zhejiang Provincial Hospital of Traditional Chinese Medicine, Hangzhou, Zhejiang, China; 3 Medical Records and Statistics Room, Zhejiang Provincial People's Hospital, Hangzhou, Zhejiang, China; 4 Medical Records and Statistics Room, the Second Affiliated Hospital of Zhejiang Chinese Medical University, Hangzhou, Zhejiang, China; Aga Khan University, KENYA

## Abstract

**Background:**

Many comparability problems appear in the process of the performance assessment of medical service. When comparing medical evaluation indicators across hospitals, or even within the same hospital, over time, the differences in the population composition such as types of diseases, comorbidities, demographic characteristics should be taken into account. This study aims to introduce a standardization technique for medical service indicators and provide a new insight on the comparability of medical data.

**Methods:**

The medical records of 142592 inpatient from three hospitals in 2017 were included in this study. Chi-square and Kruskal-Wallis tests were used to explore the compositions of confounding factors among populations. The procedure of stratified standardization technique was applied to compare the differences of the average length of stay and the average hospitalization expense among three hospitals.

**Results:**

Age, gender, comorbidity, and principal diagnoses category were considered as confounding factors. After correcting all factors, the average length of stay of hospital A and C were increased by 0.21 and 1.20 days, respectively, while that of hospital B was reduced by 1.54 days. The average hospitalization expenses of hospital A and C were increased by 1494 and 660 Yuan, whilst that of hospital B was decreased by 810 Yuan.

**Conclusions:**

Standardization method will be helpful to improve the comparability of medical service indicators in hospital administration. It could be a practical technique and worthy of promotion.

## Introduction

Healthcare systems across the world are facing the challenges of meeting growing demand, as well as increasing productivity, reducing costs and improving outcomes[1–3]. How to fairly allocate the scarce medical resources in an efficient and effective manner to meet the medical needs of the population while at the same time curb the excessive growth of medical costs is one of the major challenges for governments at all levels[4–6]. To solve this dilemma, various reimbursement mechanisms and medical quality evaluation indicators were introduced[7–9], for instance, diagnosis-related group (DRG), a patient classification system that standardizes prospective payment to hospitals and encourages cost containment initiatives which firstly adopted by the US Medicare Programme as the currency for reimbursing hospitals on a prospective, per-case basis[10]. As a hospital reimbursement and performance monitoring tool, DRG now has been introduced and indigenized in several countries [8, 11, 12]. Chinese health regulator has also actively explored the feasibility of DRG in China and piloted in some areas. Besides, in order to alleviate the problems of biased resource allocation and high patient flows to large hospitals, China implemented a hierarchical medical system[13]. It’s a two-way referral system that enables the basic hospitals to treat common diseases, and patients with intractable diseases are transferred to higher-level hospitals. Optimizing the average length of stay (ALOS) and controlling the average hospitalization expense(AHE) were cited as high priorities for health service providers, behind these policies, and were considered as two important efficiency indicators to assess the medical quality and management level of many health systems[14–16].

However, there are differences in rates of some phenomena between populations. They are usually confounded by the population compositions which cannot be directly compared [17, 18]. Similarly, comparability problems also exist in the assessing of medical services performance. For instance, the costs of surgical patients are higher than those of non-operative patients who have the same disease. Meanwhile, the length of hospital stays and costs for critical patients are usually higher than those of mild patients. When evaluating the medical service indicators among hospitals, disease interference is inevitable as long as there exist attribute and severity differences, which will eventually result in the medical variance. And this difference can even be caused by unreasonable medical expenses. Simply comparing the values without considering the actual condition of the patients is unfair to those hospitals with more critical patients and will dampen their enthusiasm.

Therefore, to improve the evaluation quality and make the medical service indicators results more comparable among hospitals, and among different time periods of the same hospital, the details of population composition such as types of diseases, comorbidities, demographic characteristics, etc. should be taken into account[4, 19]. DRG-based payment approach can control the costs, reduce the care intensity and shorten the hospital stays by grouping similar patients. However, it's a composite indicator which is not applicable in the assessment of single medical service indicators. Thus, it is imperative for us to find a more appropriate and objective method for the comparation of medical indicators. Standardization method is a commonly used technique for adjusting the confounding effects of population composition to enhance the comparability of indicators among multiple populations [20, 21]. The purpose of this study is to introduce a specific standardization technique for medical service indicators in hospital management by using the first-hand clinical data from three general hospitals. ALOS and AHE, the most commonly used indicators, were taken as example for presentation. Altogether, this study identified the existing needs for the assessment of medical service utilization and provided a new insight for financial reimbursement.

## Materials and methods

### Study design and data source

The study began on February 1, 2018. A total of 160164 inpatient medical records in 2017 were collected from three tertiary general hospitals, retrospectively. Patients who discharged from the emergency medical department or less than 18 years old were excluded. To protect patients’ privacy, their identities were concealed, and only medical record numbers were used. This study was in conformity with the “Ethics review methods for biomedical research involving human” promulgated by the Ministry of Health of The People's Republic of China and was performed in according to the Helsinki Declaration. The protocol has been approved by the Ethics Committee of the First Affiliated Hospital of Zhejiang Chinese Medical University.

### Measurement of variables

The data set used in this research included the individual patient variables of age, sex, date of admission and discharge, the principal diagnosis and disease code (first listed diagnosis), the number of comorbidities, admission type, length of stay, and total expenses. The principal diagnoses were coded at discharge according to the International Classification of Disease, Tenth Revision (ICD-10). There was no missing data in this analysis.

In order to facilitate the comparison of internal diseases among three hospitals, the principal diagnoses were divided into 22 categories according to ICD10 codes. The categories are as shown in Table 1.

**Table 1:** The principal diagnoses category and ICD10 code.

The principal diagnoses category	ICD10 code
Certain infectious and parasitic disease	A00-B99
Neoplasms	C00-D48
Diseases of the blood and blood-forming organs and certain disorders involving the immune mechanism	D48-D89
Endocrine, nutritional and metabolic diseases	E00-E90
Mental and behavioural disorder	F00-F99
Diseases of the nervous system	G00-G99
Diseases of the eyes and adnexa	H00-H59
Diseases of the ear and mastoid process	H60-H95
Diseases of the circulatory system	I00-I99
Diseases of the respiratory system	J00-J99
Diseases of the digestive system	K00-K93
Diseases of the skin and subcutaneous tissue	L00-L99
Diseases of the musculoskeletal system and connective tissue	M00-M99
Diseases of the genitourinary system	N00-N99
Pregnancy, childbirth and the puerperium	O00-O99
Certain conditions originating in the perinatal period	P00-P96
Congenital malformations, deformations and chromosomal abnormalities	Q00-Q99
Symptoms, signs and abnormal clinical and laboratory findings, not elsewhere classified	R00-R99
Injury, poisoning and certain other consequences of external causes	S00-T98
External causes of morbidity and mortality	V01-Y98
Factors influencing health status and contact with health services	Z00-Z99
Codes for special purposes	U00-U99

### Statistical analysis

Standardized indicators are more comparable, which means, it’s important to distinguish the differences between observed indicators into true differences and the differences caused by the component effects of the confounding factors[20, 22].

Suppose there is a medical service indicator (*T*) that needs to compare among three hospitals, recorded as *T*_1_, *T*_2_ and *T*_3_. The differences originated from confounding factors (e.g. age, disease). The algebraic expression for computing a standardized indicator with two confounding factors is shown as follows.

$$T^{\prime }=\sum N_{ij}T_{ij}/\sum N_{ij}$$

Here, the sum of discharges from three hospitals was used as the standard population. *N*_*ij*_ denotes the standard population in the *ij*th category of confounding factor (*i* = 1,2,3…,*i*; *j* = 1,2,3,…,*j*); *T*_*ij*_ is the crude value in the *ij*th category; *T*′ denotes standardized value. The calculation process is shown in Table 2.

**Table 2:** Stratified standardization method of medical service indicator among three hospitals.

Factor A(*i*)	Factor B(*j*)	Standard population (*N*_*ij*_)	Hospital A(*N*_1_)		Hospital B(*N*_2_)		Hospital C(*N*_3_)	
			Indicator (*T*_*ij*1_)	Expected (*N*_*ij*_*T*_*ij*1_)	Indicator (*T*_*ij*2_)	Expected (*N*_*ij*_*T*_*ij*2_)	Indicator (*T*_*ij*3_)	Expected (*N*_*ij*_*T*_*ij*3_)
*i* = 1	*j* = 1	*N*_11_	*T*_111_	*N*_11_ ∙ *T*_111_	*T*_112_	*N*_11_ ∙ *T*_112_	*T*_113_	*N*_11_ ∙ *T*_113_
	*j* = 2	*N*_12_	*T*_121_	*N*_12_ ∙ *T*_121_	*T*_122_	*N*_12_ ∙ *T*_122_	*T*_123_	*N*_12_ ∙ *T*_123_
	*j* = 3	*N*_13_	*T*_131_	*N*_12_ ∙ *T*_131_	*T*_132_	*N*_12_ ∙ *T*_132_	*T*_133_	*N*_12_ ∙ *T*_133_
	…	…	…	…	…	…	…	…
*i* = 2	*j* = 1	*N*_21_	*T*_211_	*N*_21_ ∙ *T*_211_	*T*_212_	*N*_21_ ∙ *T*_212_	*T*_213_	*N*_21_ ∙ *T*_213_
	*j* = 2	*N*_22_	*T*_221_	*N*_22_ ∙ *T*_221_	*T*_222_	*N*_22_ ∙ *T*_222_	*T*_223_	*N*_22_ ∙ *T*_223_
	*j* = 3	*N*_23_	*T*_231_	*N*_23_ ∙ *T*_231_	*T*_232_	*N*_23_ ∙ *T*_232_	*T*_233_	*N*_23_ ∙ *T*_233_
	…	…	…	…	…	…	…	…
…	…	…	…	…	…	…	…	…
Total		∑*N*_*ij*_	*T*_1_	∑*N*_*ij*_*T*_*ij*1_	*T*_2_	∑*N*_*ij*_*T*_*ij*2_	*T*_3_	∑*N*_*ij*_*T*_*ij*3_
Standardized indicator		—	$T_{1}^{\prime }=\sum N_{i}T_{ij1}/\sum N_{ij}$		$T_{2}^{\prime }=\sum N_{i}T_{ij2}/\sum N_{ij}$		$T_{3}^{\prime }=\sum N_{i}T_{ij3}/\sum N_{ij}$	

In this study, the ALOS and AHE were standardized according to confounding factors stratified and compared among three hospitals. According to literatures, ALOS and AHE were associated with age, females, patients with more comorbidities, patients with a higher DRG weight and the incentives of the financing system [23–25]. Hence, age, gender, comorbidity and principal diagnoses category were considered as confounding factors. *T* test, Chi-square and Kruskal-Wallis tests were used to verify these factors. Statistical analyses were performed using the Statistical Package for Social Sciences (SPSS for windows, version 18.0; Chicago, Illinois, USA). Statistical significance was set at *P*<0.05 (two-tailed).

## Results

### Clinical and demographic characteristics

Totally 160164 discharged patients were collected, 17572 cases were excluded according to exclusion criteria, and 142592 cases were analyzed. As shown in Table 3, the ALOS and AHE in total were 11.75 days and 16341 Yuan, respectively. The ALOS of three hospitals from low to high was hospital C (10.55 days) < hospital A (11.50 days) < and hospital B (16.13 days). Whereas, the AHE was hospital B (11028 Yuan) < hospital C (16663 Yuan) < hospital A (17299 Yuan).

**Table 3:** Total number of discharges, ALOS and AHE of three hospitals’ discharges.

	Hospital A	Hospital B	Hospital C	Total
Discharges	79,470	17,119	46,003	142,592
ALOS	11.50	16.13	10.55	11.75
AHE(CNY)	17,299	11,028	16,663	16,341

ALOS, average length of stay; AHE, average hospitalization expense; Total, the common standard

Table 4 provided the baseline characteristics of patients from each hospital. The results indicated that the population compositions, e.g. gender, age, comorbidities and disease classification, were significantly different among three hospitals. The proportion of female were higher than that of male in all three hospitals. The median age of patients in hospital A, hospital B, and hospital C were 57 years (IQR 41~72), 62 years (IQR 45~78), and 53 years (IQR 39~67), respectively. Patients in hospital B were older than hospital A and hospital C. The percentage of comorbidities in hospital B (79.1%) was also higher than the other hospitals. Meanwhile, there were some differences in the disease composition among three hospitals. The highest percentage of diseases diagnoses category in hospital A, B, and C was I00-I99 (17.9%), I00-I99 (17.4%), and C00-D48 (30.0%), respectively.

**Table 4:** Characteristics of discharges among three hospitals.

	Hospital A (n = 79470)	Hospital B (n = 17119)	Hospital C (n = 46003)	*P* value
Gender, n(%)				<0.001
male	38118(48.0)	8345(48.7)	20641(44.9)	
female	41352(52.0)	8774(51.3)	25362(55.1)	
Age				<0.001
mean(SD)	56.2(19.7)	60.4(20.1)	53.1(18.5)	
median(Q_L_~Q_U_)	57(41~72)	62(45~78)	53(39~67)	
No. of comorbidities, n(%)				<0.001
0	25450(32.0)	3572(20.9)	15805(34.4)	
≥1	54020(68.0)	13547(79.1)	30198(65.6)	
The principal diagnoses category, n(%)				<0.001
A00-B99	1252(1.6)	176(1.0)	909(2.0)	
C00-D48	8556(10.8)	991(5.8)	13810(**30.0**)	
D50-D89	346(0.4)	174(1.0)	1246(2.7)	
E00-E90	2719(3.4)	732(4.3)	1916(4.2)	
F00-F99	261(**0.3**)	210(1.2)	188(**0.4**)	
G00-G99	2864(3.6)	400(2.3)	1026(2.2)	
H00-H59	1759(2.2)	114(0.7)	813(1.8)	
H60-H95	459(0.6)	95(0.6)	724(1.6)	
I00-I99	14238(**17.9**)	2949(**17.2**)	4552(9.9)	
J00-J99	3853(4.9)	1360(7.9)	3091(6.7)	
K00-K93	7225(9.1)	1582(9.2)	3668(8.0)	
L00-L99	624(0.8)	207(1.2)	416(0.9)	
M00-M99	3051(3.8)	1669(9.8)	3300(7.2)	
N00-N99	5472(6.9)	1218(7.1)	3065(6.7)	
O00-O99	8012(10.1)	1145(6.7)	2687(5.8)	
Q00-Q99	455(0.6)	30(**0.2**)	198(0.4)	
R00-R99	1188(1.5)	513(3.0)	778(1.7)	
S00-T98	3885(4.9)	2143(12.5)	2496(5.4)	
Z00-Z99	13251(16.7)	1411(8.2)	1120(2.4)	

SD, standard deviation; Q_L_, lower quartile(P25); Q_U_, upper quartile(P75).

### Standardization of ALOS

The total number of discharged patients in all three hospitals was taken as a common standard to facilitate comparisons. Assume that the principal diagnoses category played a crucial role in hospitalization days and expense among all confounding factors. Firstly, the standard population were stratified by the principal diagnoses category, and the standardized ALOS was calculated. Then, the ALOS was adjusted by comorbidities and the principal diagnoses category. The standardized processes were shown in Tables 5–7. Similarly, the remaining confounding factors were adjusted in the same way. Table 7 presented the adjusted ALOS in each step. It changed every time after adjusting each of the confoundings. After correcting all factors, the ALOS of three hospitals from low to high was hospital A (11.71 days) < hospital C (11.75 days) < and hospital B (14.59 days). In other words, the ALOS of hospital A and C were increased by 0.21 and 1.20 days, respectively, whilst that of hospital B was reduced by 1.54 days.

**Table 5:** The standardized ALOS by disease category among three hospitals.

**Disease category**	**Standard discharges**	**Hospital A**		**Hospital B**		**Hospital C**	
		ALOS	Expected discharged bed day	ALOS	Expected discharged bed day	ALOS	Expected discharged bed day
A00-B99	2337	9.46	22112	20.38	47630	8.46	19778
C00-D48	23357	13.03	304389	19.38	452739	10.54	246077
D50-D89	1766	11.64	20564	24.25	42820	10.79	19053
E00-E90	5367	11.19	60034	15.05	80769	13.23	71018
F00-F99	659	18.73	12344	17.22	11350	16.29	10733
G00-G99	4290	26.60	114133	17.11	73391	13.34	57213
H00-H59	2686	4.48	12031	5.28	14184	4.77	12812
H60-H95	1278	8.39	10725	9.94	12699	10.22	13064
I00-I99	21739	18.01	391543	21.23	461569	14.34	311820
J00-J99	8304	13.46	111793	17.48	145192	10.97	91129
K00-K93	12475	9.13	113914	14.48	180651	9.32	116247
L00-L99	1247	8.21	10238	19.00	23693	12.93	16127
M00-M99	8020	13.15	105459	14.60	117061	10.83	86866
N00-N99	9755	8.03	78313	12.20	119046	8.11	79065
O00-O99	11844	2.87	33981	6.75	79981	4.09	48469
Q00-Q99	683	9.73	6644	7.77	5305	10.44	7134
R00-R99	2479	8.88	22021	12.01	29777	8.71	21604
S00-T98	8524	13.75	117219	20.08	171136	14.21	121095
Z00-Z99	15782	8.20	129442	11.71	184731	9.64	152184
Total	142592	11.50	1676898	16.13	2253725	10.55	1501488
Standardized ALOS		11.76		15.81		10.53	

ALOS, average length of stay.

**Table 6:** The standardized ALOS by disease category and comorbidity among three hospitals.

**comorbidity**	**Disease category**	**Standard discharges**	**Hospital A**		**Hospital B**		**Hospital C**	
			ALOS	Expected discharged bed day	ALOS	Expected discharged bed day	ALOS	Expected discharged bed day
Single disease	A00-B99	820	6.05	4962	22.40	18368	7.12	5836
	C00-D48	10010	9.40	94110	12.75	127614	6.99	69951
	D50-D89	508	7.30	3708	13.25	6731	8.83	4484
	E00-E90	669	6.75	4517	8.61	5759	7.12	4761
	F00-F99	93	7.37	685	11.21	1043	4.50	419
	G00-G99	493	11.84	5837	9.10	4488	5.27	2596
	H00-H59	355	3.68	1305	4.10	1456	4.16	1476
	H60-H95	549	8.00	4392	6.61	3630	9.75	5351
	I00-I99	2308	8.12	18741	10.60	24465	7.22	16654
	J00-J99	1698	8.54	14502	8.68	14738	6.35	10784
	K00-K93	4017	6.08	24435	8.85	35534	6.26	25134
	L00-L99	546	5.76	3143	7.82	4268	10.20	5571
	M00-M99	2804	9.06	25416	11.13	31195	6.24	17489
	N00-N99	3388	5.96	20203	5.32	18020	5.55	18811
	O00-O99	6229	1.40	8705	5.65	35164	3.22	20080
	Q00-Q99	253	7.88	1994	8.00	2024	8.55	2162
	R00-R99	701	6.03	4225	7.33	5138	4.92	3446
	S00-T98	2579	10.18	26247	15.83	40829	10.13	26119
	Z00-Z99	6807	5.31	36137	7.67	52226	7.27	49465
With comorbidities disease	A00-B99	1517	11.60	17602	19.46	29525	9.07	13757
	C00-D48	13347	15.54	207417	20.85	278232	13.54	180742
	D50-D89	1258	12.56	15795	24.78	31170	11.86	14920
	E00-E90	4698	11.97	56228	15.48	72730	14.01	65797
	F00-F99	566	19.62	11107	18.55	10501	19.08	10799
	G00-G99	3797	27.57	104665	18.46	70111	16.08	61040
	H00-H59	2331	4.60	10713	5.70	13292	4.86	11324
	H60-H95	729	8.67	6323	10.71	7811	10.64	7758
	I00-I99	19431	19.31	375304	22.01	427595	15.13	293909
	J00-J99	6606	14.44	95361	18.40	121546	12.98	85719
	K00-K93	8458	10.88	92009	16.12	136371	10.51	88899
	L00-L99	701	10.66	7475	24.84	17412	14.69	10299
	M00-M99	5216	14.88	77588	16.09	83944	14.20	74043
	N00-N99	6367	9.38	59721	14.02	89250	9.29	59120
	O00-O99	5615	5.13	28791	7.14	40085	4.68	26266
	Q00-Q99	430	11.00	4732	7.56	3252	11.14	4789
	R00-R99	1778	10.17	18091	13.06	23224	10.38	18454
	S00-T98	5945	15.43	91727	21.88	130106	15.79	93870
	Z00-Z99	8975	10.21	91678	14.54	130539	15.26	136943
Total		142592	11.50	1675593	16.13	2149384	10.55	1549034
Standardized ALOS			11.75		15.07		10.86	

ALOS, average length of stay.

**Table 7:** Results of standardized ALOS among three hospitals based on confounding factor stratification.

Hospital	Crude value(rank)	^a^Adjusted 1(rank)	^b^Adjusted 2(rank)	^c^Adjusted 3(rank)	^d^Adjusted 4(rank)	^e^ Difference
A	11.50(2)	11.76(2)	11.75(2)	11.72(2)	11.71(1)	0.21
B	16.13(3)	15.81(3)	15.07(3)	14.55(3)	14.59(3)	-1.54
C	10.55(1)	10.53(1)	10.86(1)	11.14(1)	11.75(2)	1.20

ALOS, average length of stay

^a^: Disease category

^b^: Disease category and comorbidity

^c^: Disease category, comorbidity and age

^d^: Disease category, comorbidity, age and gender

^e^: Standardized ALOS difference between adjusted 4 and crude value.

### Standardization of the average hospitalization expense

The calculation method of standardized AHE was the same as standardized ALOS. Its calculation process was shown in Tables 8–10. Table 10 summarized the results of AHE in adjusting confounding factors. If the compositions of all confounding factors (i.e. gender, ethnicity, age, and education in this study) were the same, the differences in standardized AHE of hospital A and C were increased by 1494 and 660 Yuan, while that of hospital B was reduced by 810 Yuan. The outcome is in accordance with ALOS.

**Table 8:** The standardized AHE by disease category among three hospitals (CNY).

**Disease category**	**Standard discharges**	**Hospital A**		**Hospital B**		**Hospital C**	
		AHE	Expected total charges	AHE	Expected total charges	AHE	Expected total charges
A00-B99	2337	11463	26788806	9593	22419431	9212	21529086
C00-D48	23357	30928	722382474	19077	445592897	18524	432659886
D50-D89	1766	18021	31825180	16840	29738850	24752	43712046
E00-E90	5367	11389	61122764	7834	42046113	11179	59995907
F00-F99	659	14798	9751802	10593	6980908	14042	9253604
G00-G99	4290	22875	98133331	12017	51545935	15186	65148920
H00-H59	2686	9042	24286077	3690	9912165	9794	26307103
H60-H95	1278	7192	9190847	3827	4891000	9296	11879699
I00-I99	21739	18283	397450185	13820	300438289	25692	558513625
J00-J99	8304	22333	185455428	18216	151266055	16003	132892803
K00-K93	12475	16184	201896389	9950	124130577	12615	157378172
L00-L99	1247	7879	9825472	9916	12364681	8592	10713677
M00-M99	8020	22190	177966378	7550	60548556	16895	135498987
N00-N99	9755	15898	155080858	6236	60835073	11398	111183448
O00-O99	11844	3700	43827716	2142	25373525	4226	50047351
Q00-Q99	683	22420	15312810	7088	4841358	17153	11715678
R00-R99	2479	10082	24994008	8573	21253044	9835	24380231
S00-T98	8524	31873	271688524	13660	116433629	29388	250507549
Z00-Z99	15782	12936	204158943	8400	132563731	12925	203983889
Total	142592	17299	2671137991	11028	1623175818	16663	2317301660
Standardized ALOS		18733		11383		16251	

AHE, average hospitalization expense.

**Table 9:** The standardized AHE by disease category and comorbidity among three hospitals (CNY).

**comorbidity**	**Disease category**	**Standard discharges**	**Hospital A**		**Hospital B**		**Hospital C**	
			AHE	Expected total charges	AHE	Expected total charges	AHE	Expected total charges
Single disease	A00-B99	820	6275	5145175	6909	5665503	6366	5220337
	C00-D48	10010	23818	238421957	10858	108693356	12637	126495346
	D50-D89	508	11371	5776558	6601	3353206	16038	8147303
	E00-E90	669	15454	10338821	4807	3216062	8608	5758840
	F00-F99	93	14931	1388580	6373	592700	3360	312453
	G00-G99	493	22302	10995049	3889	1917281	4365	2151797
	H00-H59	355	6156	2185469	1919	681313	6991	2481872
	H60-H95	549	7879	4325523	1701	933798	9205	5053589
	I00-I99	2308	17221	39745186	5546	12800335	11755	27131287
	J00-J99	1698	8660	14705312	3813	6475253	6377	10827328
	K00-K93	4017	11636	46741687	6905	27738089	9397	37747237
	L00-L99	546	4827	2635635	2724	1487243	5773	3152217
	M00-M99	2804	22144	62091420	4851	13601322	10187	28565516
	N00-N99	3388	13549	45903148	3233	10954950	7653	25927867
	O00-O99	6229	1467	9135990	1224	7623966	2709	16872292
	Q00-Q99	253	21510	5442126	10372	2624237	13525	3421815
	R00-R99	701	6601	4627221	3364	2357959	4398	3083307
	S00-T98	2579	20014	51615634	10154	26186721	19554	50430476
	Z00-Z99	6807	9331	63518847	5637	38371156	9409	64048850
With comorbidities disease	A00-B99	1517	14722	22332689	10813	16403775	10492	15916817
	C00-D48	13347	35840	478355127	20889	278809685	23513	313826144
	D50-D89	1258	19416	24425494	17333	21805046	29509	37122366
	E00-E90	4698	10671	50131763	8037	37758599	11504	54043787
	F00-F99	566	14787	8369685	11526	6523446	16572	9379683
	G00-G99	3797	22912	86997492	13394	50855213	18859	71608885
	H00-H59	2331	9461	22052767	4323	10076560	10196	23767504
	H60-H95	729	6697	4882353	4324	3152269	9375	6834513
	I00-I99	19431	18423	357973670	14422	280238178	27221	528924378
	J00-J99	6606	25036	165390401	19712	130220246	20172	133256383
	K00-K93	8458	18790	158928826	10838	91666085	13869	117302249
	L00-L99	701	10931	7662914	13670	9582676	10407	7295507
	M00-M99	5216	22210	115847048	8714	45452324	21807	113745767
	N00-N99	6367	17435	111010494	7028	44744101	13129	83591131
	O00-O99	5615	7128	40025890	2462	13826902	5248	29468289
	Q00-Q99	430	23049	9910998	4215	1812356	18479	7946172
	R00-R99	1778	11657	20726172	9742	17321199	12216	21720420
	S00-T98	5945	37440	222579672	15152	90080285	33206	197411047
	Z00-Z99	8975	15444	138613580	10345	92845372	21234	190577782
Total		142592	17299	2670956375	11028	1518448767	16663	2390568552
Standardized average charges			18731		10649		16765	

AHE, average hospitalization expense.

**Table 10:** Results of standardized AHE among three hospitals based on confounding factor stratification(CNY).

Hospital	Crude value(rank)	^a^Adjusted 1(rank)	^b^Adjusted 2(rank)	^c^Adjusted 3(rank)	^d^Adjusted 4(rank)	^e^Difference
A	17299(3)	18733(3)	18731(3)	18855(3)	18793(3)	1494
B	11028(1)	11383(1)	10649(1)	10145(1)	10218(1)	-810
C	16663(2)	16251(2)	16765(2)	17229(2)	17323(2)	660

AHE, average hospitalization expense

^a^: Disease category

^b^: Disease category and comorbidity

^c^: Disease category, comorbidity and age

^d^: Disease category, comorbidity, age and gender

^e^: Standardized AHE difference between adjusted 4 and crude value.

## Discussion

A total of 142592 discharged patients from three hospitals were analyzed in the current study. Our results implied that the differences in compositions of demographic characteristics, comorbidity and principal diagnoses category might impose a substantial impact on comparing observed outcomes among three hospitals. When comparing with the crude value, the ALOS of hospital A and C were increased by 0.21 and 1.20 days, while that of hospital B was decreased by 1.54 days. The AHE displays the same trend. That is, the standardized AHE of hospital A and C were increased but that of hospital B was reduced when compared with before.

As far as we know, standardization technique is commonly used for comparing rates, such as cure rates, death rates and birth rates, between different groups or populations[17, 18, 26]. However, there currently has been no report on the application of standardization method in hospital administration. Our findings indicated that the idea of stratified standardization can also be applied to the evaluation of medical services. Our study presented a detailed analysis and discussion of the standardization method, including the determination of common standard, identification of confounding factors, and hierarchical standardization of ALOS and AHE. The results demonstrated that after adjusting confounding factors the real differences in ALOS and AHE among three hospitals were much smaller than the original values, although the order did not change dramatically. For instance, the difference between the maximum and the minimum values of ALOS was reduced from 5.58 days to 2.88 days after adjusting disease category, comorbidity, age and gender. And the trend of AHE is consistent with that of ALOS which suggested that the adjusted medical service indicators are more reflective of the quality of care between hospitals.

Under the reform of public hospitals in China, the evaluation mechanisms of public hospitals are becoming more and more competitive. The standardization method could effectively increase the comparability of medical service indicators and has positive significance for the formulation of public hospital policy. Standardized indicators could improve the fairness of hospital assessment and reduce speculation. In order to control the average hospital expense, some medical institutions adopt unreasonable ways to reduce the expense, such as re-admission of long-term inpatients and admission of mild patients who do not require hospitalization. The impact of these opportunistic behaviors could be adjusted through standardization during assessment. On the other hand, it is conducive to promoting the implementation of hierarchical medical system, reducing the burden of large hospitals and enhancing the capacity of primary medical services. China has vigorously promoted the implementation of the hierarchical medical system to provide different levels of medical services according to the patients' conditions and to realize rational allocation of medical resources[13]. Although the hierarchical medical system has many advantages, its impact is still limited. Large hospitals are still overcrowded, while primary medical institutions are to some extent unwanted. Through the standardization of medical data, it is possible to make the problem more obvious for health department and make it easy to identify those high-level hospitals that treated a large number of patients with mild illnesses. Based on this, the government and health department can better supervise these hospitals and eventually optimize the system model to achieve the rational allocation of medical resources.

The application and continuous evaluation of clinical pathways (CP) in health-care settings benefit the institutionalization of culture of quality in hospitals[27]. The standardized method can be used to adjust the assessment indexes in each stage of CP. The process of disease diagnosis and treatment will be more normative after standardized indicators were applied. Moreover, the symptoms of inpatients are complex and diverse, the adoption of a “one-size-fits-all” approach will inevitably dampen doctors' enthusiasm. The hierarchical standardization of medical indicators is likely to promote the classification management of disease and provide direction for continuous improvement of medical quality.

The allocation of funds and health resources as well as the control of deficits of the national health system are the major and long-standing problems, which are also at the heart of health care reform. [28]. Through the standardization of the composition of medical expenses, it will be possible to find and solve the core problem of “expensive medical treatment”. Currently, DRG approach has been recognized and our standardization method could be a complement to it. It provides a standard for more precise grouping of DRGS and an objective basis for differentiated financial subsidies.

In drawing meaningful conclusions from this study, it is important to be cognizant of its limitations. Not all the confounding factors have been taken in to consideration. Only age, sex, and disease category were selected in this analysis. Some specific details of disease diagnosis, such as tumor stage, were not included as confounding factors. Notwithstanding these limitations, this study highlights an approach and some suggestions in the comparison of medical indicator evaluation. In addition, these standardized indicators no longer reflect reality, they are only a reference level for comparisons between hospitals and departments within hospitals.

## Conclusions

There are many comparability problems in the assessment of medical service performance. In this study, by taking ALOS and AHE as examples, we introduced a specific technique for standardization, which will be helpful to improve the comparability of medical service indicators. In addition to the confounding factors described in this paper, other potential confounding factors may also contribute to the standardization. Our findings showed that our standardization method could be a practical technique and worthy of promotion.

## Supporting information

S1 File Data set. (XLSX)
